# Morphology-Dependent Percolation and Conductive Network Formation in Polymer Nanocomposites with Multi-Shaped Nanofillers

**DOI:** 10.3390/nano16010052

**Published:** 2025-12-30

**Authors:** Chang Xu, Yixuan Zhao, Hualong Zhang

**Affiliations:** 1 School of Design and Art, Shanghai Dianji University, Shanghai 200240, China; zhanghualong@st.sdju.edu.cn; 2 College of Mechanical Engineering, Donghua University, Shanghai 201620, China; 1239101@mail.dhu.edu.cn

**Keywords:** polymer nanocomposites, nanofiller morphology, hybrid fillers, percolation behavior, conductive network formation, molecular dynamics simulation

## Abstract

The electrical performance of polymer nanocomposites strongly depends on the morphology of nanofillers and the structure of the resulting conductive networks. To elucidate the mechanisms governing conductive network formation in multi-morphology nanofiller systems, a ternary coarse-grained model composed of rod-, Y-, and X-shaped nanofillers is constructed. The effects of nanofiller volume fraction (VF) and nanofiller composition ratios on percolation behavior are systematically investigated. By incorporating an efficient cKDTree-based neighbor search method, conductive networks are identified and their topological characteristics are quantified with high computational efficiency. The results demonstrate that nanofiller morphology ratios play a crucial role in controlling local structural evolution and the percolation threshold. Statistical analyses of the main cluster size (MCs) and the number of clusters (Nc) further reveal the synergistic and competitive effects among different filler morphologies. The combination of filler morphologies is shown to be a key factor in determining the percolation threshold and network topology. The multi-morphology simulation framework together with structural characterization approach proposed in this work provide theoretical guidance for the rational design of high-performance conductive polymer nanocomposites.

## 1. Introduction

Conductive composite materials based on soft organic matrices have been extensively investigated for their tunable electrical properties, including systems containing oligomers, polymers, and small-molecule organic semiconductors. Oligomer-based matrices, with their shorter chain length and lower entanglement, can display charge-transport and percolation behavior distinct from long-chain polymers [1]. However, in many practical applications, the continuous phase is still predominantly polymeric because polymer matrices combine mechanical robustness and processability with conductivity that can be tuned by incorporating nanoscale fillers. Among these, polymer nanocomposites have attracted considerable attention due to their excellent performance, lightweight nature, and tunable electrical conductivity, showing broad application potential in flexible electronic devices [2,3], sensors [4,5], and energy storage systems [6]. The enhancement of electrical conductivity in these materials primarily relies on the formation of three-dimensional percolating conductive networks by conductive nanofillers dispersed within the polymer matrix. The morphology, spatial distribution, and inter-filler connectivity of the nanofillers play a decisive role in governing the network formation process [7]. In particular, nanofillers with high aspect ratios or branched geometries can significantly reduce the percolation threshold by enhancing local connectivity, making a fundamental understanding of their network construction mechanisms essential for optimizing the performance of polymer nanocomposites [8,9,10].

In recent years, substantial progress has been made in elucidating the formation mechanisms of percolating conductive networks in polymer nanocomposites through both experimental investigations and multiscale simulations [11,12]. Early studies primarily focused on single-morphology nanofiller systems, in which nanofiller geometry was identified as a key factor governing percolation behavior [13,14]. Coarse-grained molecular dynamics simulations have demonstrated that increasing the dispersion in aspect ratio leads to a reduced percolation threshold, while the application of external shear fields can induce network restructuring and result in non-monotonic percolation transitions, highlighting the strong sensitivity of conductive networks to morphological parameters [15]. Beyond single-morphology systems, the effects of size polydispersity and bimodal particle distributions have also been extensively explored. Zhao et al. [16] systematically investigated conductive network structures in binary mixtures of large and small particles, revealing that an increasing fraction of large particles leads to a monotonic increase in the percolation threshold, whereas a subtle weak antagonistic effect may arise from the competition between the numerical advantage of small particles and the superior connectivity of large ones. Building upon these studies, increasing attention has been directed toward the roles of external fields and interfacial interactions in regulating percolation networks. Comparative studies on non-spherical nanofillers with different geometries under tensile and shear deformations have demonstrated that nanofillers capable of providing multiple connection directions exhibit enhanced network stability under external fields, in contrast to spherical nanofillers with limited contact orientations, thereby improving both isotropic and directional conductive performance [17]. In addition, studies on surface-functionalized spherical nanofillers have shown that enhanced filler–matrix interactions can modify percolation behavior, further highlighting the role of interfacial effects in conductive network formation [18]. Meanwhile, research has expanded from single-morphology systems to multi-morphology, multiscale, and hybrid filler systems. New modeling approaches have been proposed to predict the conductive behavior of nanofiber composites, emphasizing the dominant influence of fiber morphology and spatial distribution on network formation [19]. In polymer–carbon black systems, incorporating interfacial layer thickness, particle size disparity, and contact geometry yields tunneling conduction models that better agree with experimental observations, revealing nonlinear percolation characteristics in mixed-scale filler systems [20]. Related studies on micro-SiC whisker/carbon black hybrids further demonstrate that high aspect ratio whiskers can markedly reduce the percolation threshold, reflecting the tunability of structural synergistic effects [21]. Moreover, recent simulations have shown that the geometry and spatial arrangement of rigid nanofillers can simultaneously affect electrical percolation and thermal transport networks, highlighting the multifaceted role of nanofiller morphology under multi-physical conditions [22]. Despite these advances, recent investigations into synergistic effects in mixed-morphology systems have largely been confined to binary nanofiller combinations [23,24]. Systematic mechanistic understanding of more complex ternary or multi-morphology systems remains limited, motivating the present study [17]. As a result, the structural synergistic effects, competitive packing behaviors, and their combined influence on the percolation threshold in multi-morphology nanofiller systems remain an important scientific issue that has yet to be systematically understood.

To address these gaps, a ternary coarse-grained model composed of rod, Y, and X nanofillers is constructed. The effects of both the total nanofiller VF and nanofiller composition ratios on the formation of percolating conductive networks are systematically investigated. The nanofiller VF is treated as a key control parameter, because it directly governs the onset of electrical percolation, network topology, and the trade-off between target conductivity and filler loading that is crucial for processing and mechanical performance in practical polymer composites. By incorporating a cKDTree-based neighbor search method, tunneling contacts and filler clusters can be identified efficiently even in large-scale systems. Furthermore, the cKDTree-based strategy is systematically benchmarked against the conventional naive all-pairs method, revealing an almost 180-fold reduction in the average computational time per frame while maintaining accurate network identification and percolation statistics over a wide range of volume fractions and filler morphology combinations. This study elucidates the roles of competitive packing, cluster merging behavior, and their decisive influence on the percolation threshold while clearly distinguishing the differentiated contributions of rod, Y, and X nanofillers to conductive network formation.

## 2. Model and Simulation Methods

### 2.1. Model Construction

A coarse-grained model is employed to simulate the formation of conductive networks in polymer nanocomposites. Polymer chains are represented using a classical bead–spring model, with each chain consisting of 30 beads. The simulation system contains a total of 1400 polymer chains, ensuring that the system exhibits representative static and dynamic behaviors characteristic of polymer melts. Although the chains are shorter than real polymer chains, this level of coarse-grained model has been shown to be sufficient for capturing essential long-chain conformational and dynamical features. Three types of nanofillers with distinct morphologies are considered, namely rod, Y, and X fillers. Each nanofiller is constructed by connecting 10 beads, with bead diameter and mass identical to those of the polymer beads. This unified bead representation facilitates consistent modeling of filler–polymer and filler–filler interactions.

To systematically investigate the cooperative effects among different filler morphologies, three representative composition ratios are considered, namely 1:1:1, 1:1:3, and 1:1:8. These ratios are chosen to span a progression from a completely symmetric ternary mixture to moderately asymmetric and strongly asymmetric mixtures while keeping the number of each filler type sufficiently large for reliable statistics at a fixed total filler number. By permuting which morphology corresponds to the dominant component in the asymmetric cases, each filler type can in turn act as the majority phase or as a minority co-filler, allowing the morphology-dependent synergistic and competitive effects to be systematically compared within a manageable computational cost. Considering that rod, Y, and X fillers can each serve as the dominant component at higher loadings, a total of seven distinct compositional systems are examined. This design enables direct comparison between systems with evenly mixed fillers and those dominated by a specific filler morphology, allowing the influence of filler composition on conductive network formation to be clearly identified. The filler VF differ among the systems after equilibration due to the use of different nanofiller loadings. The corresponding values are summarized in Table 1. This systematic combinatorial design allows conductive network formation to be compared across different VFs and filler ratios within a unified simulation framework, providing a reliable computational basis for elucidating synergistic effects in ternary nanofiller systems.

### 2.2. Interaction Potentials and Parameter Settings

In the present coarse-grained modeling framework, all interactions are described using simplified potential energy functions, ensuring that the system captures the essential physical characteristics of polymer nanocomposites while remaining computationally tractable. Polymer–polymer interactions are modeled using a truncated and shifted Lennard–Jones potential, expressed as

(1) $$U(r)=\left\{\begin{array}{c} \begin{array}{cc} 4\varepsilon \left[{\left(\frac{\sigma }{r}\right)}^{12}-{\left(\frac{\sigma }{r}\right)}^{6}\right] & r<r_{\mathrm{cutoff}} \end{array}\\ \begin{array}{cc} 0 & r\geq r_{\mathrm{cutoff}} \end{array} \end{array}\right.$$

where *ε* denotes the interaction strength, *σ* is the characteristic length scale, and *r*_cutoff_ is the cutoff distance. In real polymer systems, *ε* typically falls in the range of approximately 2.5–4.0 kJ·mol^−1^. For the coarse-grained polymer chain model employed here, the persistence length is approximately 0.676σ. Given that the persistence length of real polymers usually lies between 0.35 and 0.76 nm, the length scale σ can be reasonably mapped to approximately 1 nm. Accordingly, the diameter of each coarse-grained bead is set to 1 nm, which roughly corresponds to five polyethylene repeat units, with an associated bead mass of approximately 140 g·mol^−1^. Based on the reduced Lennard–Jones units, the characteristic time scale is defined as

(2) $$\tau =\sigma (m/e{)}^{1/2}$$

This yields a time scale of approximately 10 ps, thereby ensuring that the simulations are physically meaningful. The Lennard–Jones potential is truncated and shifted such that both the interaction energy and force vanish at *r* = *r*_cutoff_. The interaction strength and cutoff distance for polymer–polymer interactions are set to *ε*_pp_ = 1.0 and *r*_pp_ = 2 × 2^1/6^*σ*, respectively. For nanofiller–nanofiller interactions, the parameters are chosen as *ε*_nn_ = 1.0 and *r*_nn_ = 1.12*σ*, corresponding to short-range interactions that prevent excessive filler overlap. The polymer–nanofiller interactions are characterized by an interaction strength of *ε*_pn_ = 1.0 and *r*_pn_ = 2.5*σ*, which is introduced to model weak attractive interactions between the polymer matrix and nanofillers.

The bonded interactions connecting adjacent beads within both polymer chains and nanofillers are described using a finitely extensible nonlinear elastic (FENE) potential, given by

(3) $$U_{\mathrm{FENE}}(r)=-0.5k{R_{0}}^{2}\ln\left[1-{\left(\frac{r}{R_{0}}\right)}^{2}\right]$$

where the spring constant is set to 30*ε*/*σ*^2^, and the maximum bond extension is *R*_0_ = 1.5*σ*. These parameters ensure sufficient bond stiffness while avoiding high-frequency vibrational modes and preventing chain crossing. To maintain the prescribed geometries of the nanofillers, angular interactions are introduced through a bending potential of the form

(4) $$U_{\mathrm{angle}}(\theta )=K{\left(\theta -\theta_{0}\right)}^{2}$$

where *θ* denotes the bending angle formed by three consecutive filler beads. The bending stiffness is set to *K* = 1000, enforcing near-rigid filler geometries. The equilibrium angle *θ*_0_ is chosen as 180° for rod fillers, 120° for Y fillers, and 90° or 180° for X fillers, depending on the specific arm configuration. As no specific polymer chemistry is targeted in this study, reduced Lennard–Jones units are employed with unified values of *σ* and *ε*, such that all reported quantities are expressed in dimensionless form.

### 2.3. Simulation Protocol and Equilibration

Following established simulation protocols [7], each system is first compressed and equilibrated under the isothermal–isobaric (NPT) ensemble for 5000 *τ*. During this stage, the temperature and pressure are maintained at T* = 1.0 and P* = 0.0 using Nose–Hoover thermostat and barostat, respectively. This procedure ensures efficient relaxation of the system density and removal of unfavorable initial overlaps. Subsequently, the systems are further equilibrated for 50,000 *τ* under the canonical (NVT) ensemble at T* = 1.0. During the equilibration process, the time evolution of the total energy, pressure, and polymer bead number density was monitored to ensure that all systems reached stationary plateaus. After the NPT and NVT stages, the translational motion of each polymer chain by tracking the displacement of its center of mass was evaluated. Every chain was verified to have diffused over a distance exceeding twice its radius of gyration. Since the radius of gyration represents the typical spatial extent of a single chain, a center-of-mass displacement larger than twice this length implies that the chain has migrated over a length scale significantly larger than its own size, has sampled statistically independent local environments, and has effectively lost memory of its initial configuration. This criterion is therefore commonly taken as evidence that polymer chains are fully relaxed and that equilibrium has been achieved for all systems. The final number density of polymer beads converges to approximately 0.85 in reduced Lennard–Jones units, which lies in the typical range of bead number densities used for coarse-grained bead–spring polymer melt [16,25]. The equilibrated configurations are then used as initial states for production runs, during which both structural and dynamical properties are analyzed. Periodic boundary conditions are applied in all three spatial directions to minimize finite-size effects. Long production simulations are performed, and system configurations are recorded at regular intervals, yielding 10,000 statistically independent equilibrium configurations for subsequent analysis. The sampling interval between consecutive configurations is set to 10*τ*, which is sufficient to ensure statistical independence. All molecular dynamics simulations are carried out using the Large-scale Atomic/Molecular Massively Parallel Simulator (LAMMPS) [26]. The equations of motion are integrated using the velocity–Verlet algorithm with a time step of Δ*t* = 0.001 *τ*, where time is expressed in reduced Lennard–Jones units.

### 2.4. Conductive Network Identification and Data Analysis

In this study, inter-filler contacts are adopted as the physical criterion for defining conductive connections between nanofillers. For the three types of fillers, a conductive edge is assigned between a pair of nanofillers if the distance between any bead pair belonging to the two fillers is smaller than the tunneling distance, which is set to 1.0 *σ*. Based on this criterion, the entire system can be abstracted as an undirected graph G = (V, E), where the vertex set *V* represents nanofillers and the edge set E corresponds to potential conductive connections. To efficiently identify all interacting filler pairs in large-scale systems, a cKDTree-based neighbor search method is employed to perform rapid neighbor searches, significantly reducing the computational complexity. In this approach, the three-dimensional coordinates of all filler beads are first organized into a hierarchical spatial tree structure, which recursively partitions the simulation box into local bounding volumes. For each bead, only candidates within a spherical query radius equal to the tunneling distance are retrieved from the tree and subjected to explicit distance evaluation, thereby reducing the computational complexity of neighbor detection from the quadratic scaling of the naive all-pairs search to approximately O(N log N). Based on the detected tunneling contacts, the filler–filler connectivity is then represented as an undirected graph, in which each node corresponds to a nanofiller and an edge is assigned whenever a contact is present. Subsequently, the union–find algorithm is applied to this graph to determine its connected components, yielding the size and spatial distribution of all filler clusters. If a cluster forms a continuous network spanning the simulation box along all three orthogonal directions, the system is classified as three-dimensionally conductive. During the entire production stage, the above procedure is applied to 10,000 equilibrated configurations. The conductive probability is defined as the ratio of the number of configurations exhibiting three-dimensional percolation to the total number of sampled configurations. The evolution of conductive probability as a function of filler VF is then used to determine the percolation threshold and to elucidate synergistic effects arising from different filler morphology ratios. Following previous coarse-grained molecular dynamics studies on conductive polymer nanocomposites [7,23] the percolation threshold in our finite-size systems is defined as the nanofiller volume fraction at conductive probability is 0.5. This commonly adopted numerical criterion provides an effective threshold parameter for quantitatively comparing the influence of different filler morphology ratios on conductive network formation.

For data analysis, the radial distribution functions (RDFs) of the three nanofiller types are first calculated to characterize their spatial distributions and possible aggregation tendencies. To quantitatively describe the development of conductive networks, the cluster analysis described above is further used to compute MCs and Nc for each configuration. Here, MCs serves as a measure of network connectivity, while Nc reflects the degree of dispersion of fillers within the system.

## 3. Results and Discussion

### 3.1. Influence of Neighbor Search Strategy on Network Identification Efficiency

**Figure 1:**
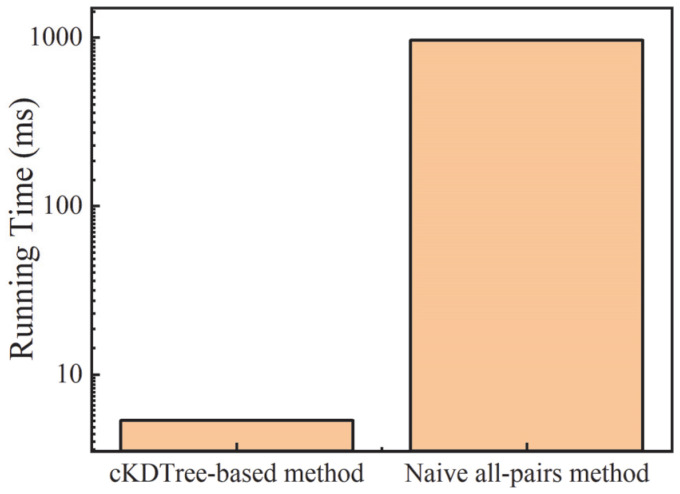
The comparison of cost time by cKDTree-based method and naive all-pairs method.

A critical step in identifying conductive filler networks is the detection of all filler pairs that satisfy the tunneling criterion. For large-scale systems containing thousands to tens of thousands of filler beads, the efficiency of the neighbor search algorithm directly determines the feasibility and reliability of percolation analysis. In this paper, a cKDTree-based neighbor search strategy was employed and systematically compared with the conventional naive all-pairs method [27]. In the naive all-pairs method, distances between all possible bead pairs must be explicitly evaluated, leading to a computational cost that scales quadratically with the number of particles. For the systems considered here, this resulted in an average computational time of 964.63 ms per simulation frame, rendering large-scale statistical analysis computationally prohibitive. By contrast, the cKDTree-based method constructs a spatial indexing structure and restricts distance evaluations to particles located within a local neighborhood. This approach significantly reduces the number of distance calculations required for each frame. The average computational time per frame using the cKDTree-based method is reduced to 5.33 ms under identical conditions as shown in Figure 1, corresponding to an approximately 180-fold improvement in computational efficiency relative to the naive all-pairs method. These results demonstrate that the cKDTree-based neighbor search is essential for efficient and scalable identification of conductive networks in large nanofiller systems, enabling reliable percolation statistics without compromising computational accuracy.

By adopting the cKDTree-based method, the total computational time for the entire dataset is reduced to approximately 50 s, which makes it feasible to systematically explore a broad parameter space spanning multiple filler VFs and compositional ratios. Moreover, the advantage of the cKDTree-based approach becomes even more pronounced in the vicinity of the percolation threshold. Filler clusters grow rapidly and large connected components emerge leading the naive all-pairs method to perform an excessive number of redundant pairwise distance checks within dense clusters, thereby further increasing its computational cost. In contrast, the localized query structure of the cKDTree-based method maintains stable performance even in highly connected and spatially dense regions, ensuring consistent efficiency across the entire percolation transition.

### 3.2. RDF Analysis of Fillers with Different Morphologies

RDF is a fundamental structural descriptor for characterizing the spatial organization of nanofillers in composite systems. By comparing the RDFs with different morphologies, insights can be obtained into their local packing characteristics and short-range ordering. Such structural information can provide a microscopic basis for understanding the mechanisms governing conductive network formation. In this paper, the spatial organization of the three types of fillers embedded in the polymer matrix is systematically examined from two complementary perspectives, which are variations in the overall filler VF and changes in the compositional ratios among different fillers. This combined analysis allows to disentangle the effects of VF and morphology on inter-filler spatial correlations.

**Figure 2:**
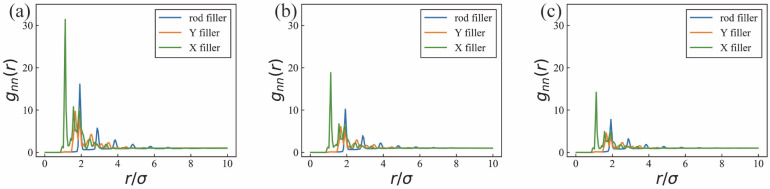
RDFs at VF of 2.87%, 4.62%, and 6.23% under a fixed equimolar composition. RDFs at different nanofiller volume fractions of (**a**) 2.87%, (**b**) 4.62%, and (**c**) 6.23% under a fixed equimolar composition.

Under an equimolar composition of rod, Y and X fillers, Figure 2 presents the RDFs of the three filler types at total filler VFs of 2.87%, 4.62% and 6.23%, respectively. As the overall filler concentration increases, a pronounced decrease in the height of the first peak of g_nn_(r) is observed, indicating a progressive reduction in short-range structural order. This behavior can be primarily attributed to the continuous reduction in free volume in the system. When the VF is 2.87%, individual fillers possess ample accessible space, which facilitates the formation of well-defined preferred inter-filler distances and leads to a sharp and prominent first RDF peak, consistent with previous simulation studies of nanofiller dispersion in polymer matrices at low loadings [7,15]. In contrast, at higher VF, the average center-to-center distance between fillers decreases, and steric crowding increasingly constrains local arrangements. As a consequence, local structural correlations are weakened, leading to a broader and flatter RDF profile.

In addition to this dependence on the total nanofiller VF, systematic differences are observed in both the positions and intensities of the first RDF peaks among the three filler morphologies. The first peak appears at the smallest *r*/σ for X fillers followed by Y fillers, while rod fillers exhibit the largest first-peak position. This ordering is directly related to geometric characteristics. X fillers feature a compact four-arm structure, which allows for smaller minimum approach distances between filler centers. Y fillers display intermediate behavior due to their three-arm geometry. In contrast, rod fillers possess a high aspect ratio that imposes more stringent geometric constraints during close approach. To minimize steric repulsion, contacts between rod fillers typically involve axial offsets or tilted configurations, causing the effective contact points to be displaced away from the geometric centers of the rods. As a result, the rod-rod center-to-center distances are systematically larger, shifting the first RDF peak to higher r/σ values compared with X and Y fillers. However, the relative heights of the first RDF peaks exhibit an inconsistent order, with X fillers showing the highest peak intensity, followed by rod fillers and then Y fillers. This hierarchy reflects differences in the degree of geometric determinacy of nearest-neighbor configurations. The compact and nearly symmetric geometry of X fillers gives rise to a relatively narrow distribution of nearest-neighbor separations, leading to a higher probability of finding neighboring fillers at similar distances and thus a more pronounced RDF peak. Rod fillers, although capable of forming close contacts, allow for a broader range of contact modes, such as end–end, end–side, and axially staggered arrangements, each of which is associated with a distinct center-to-center distance. This diversity broadens the nearest-neighbor distribution and reduces the peak intensity relative to X fillers. By contrast, the three-armed geometry of Y fillers permits highly diverse contact orientations without favoring a unique nearest-neighbor separation, resulting in a more dispersed local packing environment and the lowest first-peak intensity. Consequently, while increasing filler concentration universally weakens short-range order, the local packing specificity imposed by filler geometry governs the relative peak intensities observed.

**Figure 3:**
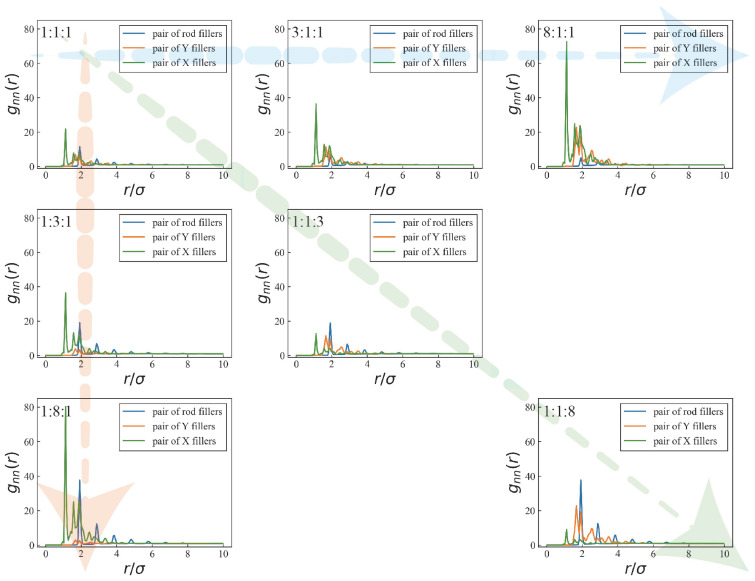
RDFs for different filler ratios at a fixed total filler number of 390.

Figure 3 illustrates the influence of filler composition on the RDFs under a fixed total filler number of 390 nanofillers, with varying ratios of rod, Y, and X fillers. All seven compositional systems exhibit pronounced morphology-dependent structural features, indicating that local inter-filler organization is governed not only by the overall filler VF but also by competitive packing effects arising from the coexistence of fillers with distinct geometries.

As the VF of rod fillers increases, a clear reduction in the peak intensity of rod-rod g_nn_(r) is observed. This trend can be attributed to the high aspect ratio of rod fillers. At low rod filler concentrations, short-range correlations between rods are primarily established through end–end or end–side point contacts, leading to relatively well-defined nearest-neighbor configurations. With increasing rod filler, excluded-volume effects become increasingly significant, favoring arrangements such as line contacts or axially staggered configurations that maximize configurational entropy. This transition toward more orientationally flexible and loosely packed structures weakens distinct nearest-neighbor correlations, resulting in a suppressed RDF peak. At the same time, increasing the rod filler VF leads to an enhancement of the RDF peak intensities for X-X and Y-Y correlations, as anisotropic crowding reduces the accessible free volume for X and Y fillers and biases them toward more confined local environments, thereby strengthening their short-range correlations.

When the VF of Y fillers increases, a progressive reduction in the peak intensity of the Y-Y RDF is observed, accompanied by a pronounced flattening of the first peak. Owing to their three-armed geometry, Y fillers permit contact configurations distributed over multiple directions, which does not favor the formation of a unique or well-defined nearest-neighbor distance. As a result, Y–Y separations span a broad and nearly continuous range, leading to intrinsically weak short-range order. With further increasing Y filler, this geometric flexibility amplifies configurational diversity, causing the Y–Y RDF peak to become flatter than those of rod–rod and X–X correlations. Meanwhile, the presence of abundant Y fillers facilitates efficient accommodation of local voids, partially relaxing excluded-volume constraints experienced by rod and X fillers and thereby enhancing their short-range correlations.

By contrast, increasing the VF of X fillers leads to a reduction and a pronounced broadening of the first peak in the X–X RDF. Due to their four-armed geometry with nearly orthogonal directions, neighboring X fillers are less likely to achieve compact contacts through symmetric or well-defined geometric configurations. The resulting geometric incompatibility produces a complex excluded-volume shape and a broad distribution of nearest-neighbor separations. As the X filler fraction increases, such configurational disorder becomes increasingly pronounced, leading to a progressive weakening of short-range correlations and a diminished first RDF peak.

### 3.3. Percolation Behavior and Conductive Network Formation

**Figure 4:**
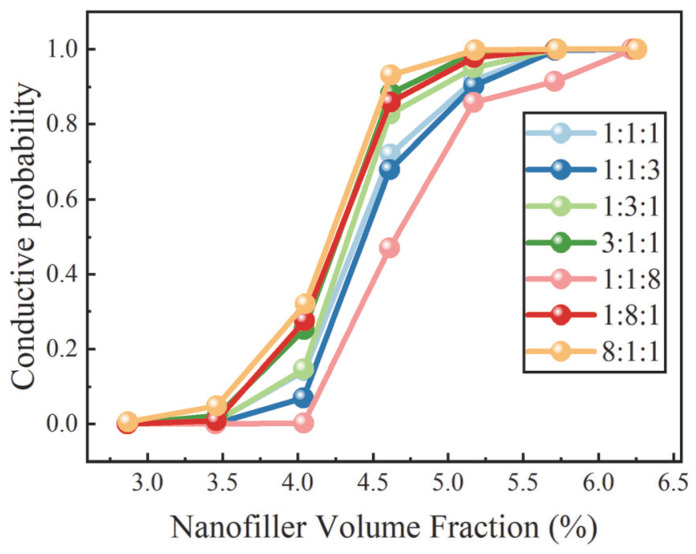
Dependence of conductive probability on nanofiller VF for different filler ratios.

Figure 4 presents the dependence of the conductive probability on the total nanofiller VF for systems with different filler composition ratios. Overall, the systems exhibit characteristic percolation behavior, with a rapid increase in conductive probability as the filler loading increases. However, the compositional ratios of different nanofillers exert a pronounced influence on both the onset of the transition region and the location of the percolation threshold.

In the low VF regime (approximately 2.87–3.46%), the conductive probability increases only gradually. In this range, the overall filler density remains insufficient to form system-spanning conductive pathways, and most connections are limited to short-range local contacts or small isolated clusters. Although increasing filler content enhances the likelihood of close inter-filler proximity, the separations between individual clusters remain relatively large, preventing these local structures from establishing effective long-range connectivity. As a result, despite the continuous increase in filler loading, the global connectivity of the system improves only slowly, giving rise to a smooth and gradual increase in conductive probability.

When the nanofiller VF enters the range of approximately 4.04–4.62%, the conductive probability exhibits a markedly accelerated increase, signaling a critical transition from local connectivity to system-spanning network formation. In this regime, the average inter-filler separation decreases substantially, enabling previously isolated small clusters to merge through the formation of bridging fillers, which rapidly expands the size of the dominant connected cluster. As percolation is a critical phenomenon, small structural perturbations near the threshold are strongly amplified. Consequently, the conductive probability rises sharply within this narrow concentration window. At this stage, the nanofiller network reaches a critical connectivity condition, such that the addition of only a small amount of filler is sufficient to establish a stable three-dimensional percolating pathway across the system. Consistent with percolation theory, crossing the critical threshold is accompanied by an abrupt growth of the largest cluster. Beyond the percolation threshold, the conductive probability gradually approaches unity. In this post-percolation regime, the conductive network is essentially fully established, and the overall connectivity becomes insensitive to local structural fluctuations, exhibiting instead a slow and steady saturation behavior.

In finite-size systems, the conductive probability increases continuously from 0 to 1 as the nanofiller VF increases. Following a commonly adopted criterion in percolation analysis, the VF at which conductive probability is 0.5 is taken as the percolation threshold. Based on this definition, Figure 4 reveals a pronounced dependence of the percolation threshold on filler morphology ratios. Among the compositions examined, the system with a ratio of 8:1:1 exhibits the lowest percolation threshold, whereas the threshold reaches its highest value when the filler ratio is 1:1:8.

This trend reflects the distinct abilities of fillers with different morphologies to construct system-spanning conductive networks. Owing to their high aspect ratio, rod fillers can establish extended effective connectivity over larger spatial distances even at relatively low concentrations, thereby participating more efficiently in the formation of percolating conductive pathways. According to excluded-volume theory, particles with higher aspect ratios exhibit lower critical percolation VF, as their excluded volume increases strongly with particle length. This enlarged excluded volume enhances the probability that rod fillers bridge local gaps and connect otherwise isolated clusters, enabling network formation across length scales and ultimately leading to global percolation.

In contrast, X fillers exhibit a relatively compact structure, resulting in more limited excluded-volume effects and contact geometries that are unfavorable for long-range connectivity. As a consequence, X fillers are less effective at establishing stable connections across multiple length scales, making the formation of system-spanning networks more difficult. When X fillers dominate the composition, inter-filler connections are largely confined to local regions, leading to a higher percolation threshold. In other words, a high fraction of X fillers tends to promote the formation of multiple small clusters rather than a single large percolating cluster, such that a substantially higher overall filler loading is required to achieve three-dimensional connectivity throughout the system.

**Figure 5:**
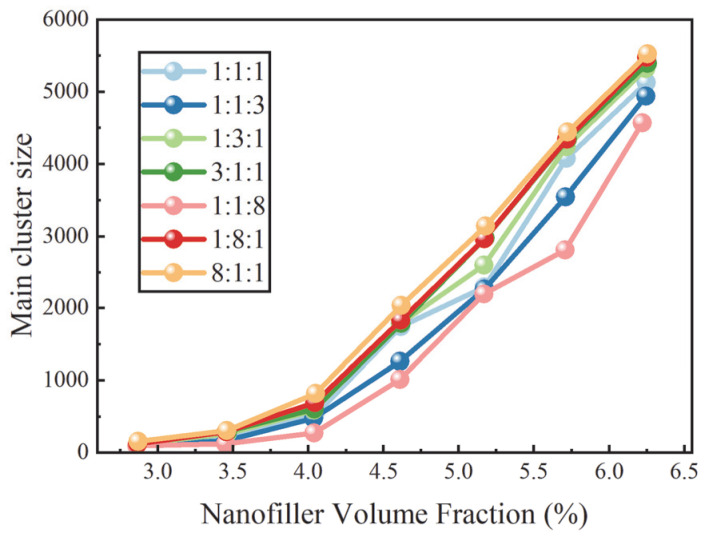
Dependence of the MCs on nanofiller VF for different filler composition ratios.

**Figure 6:**
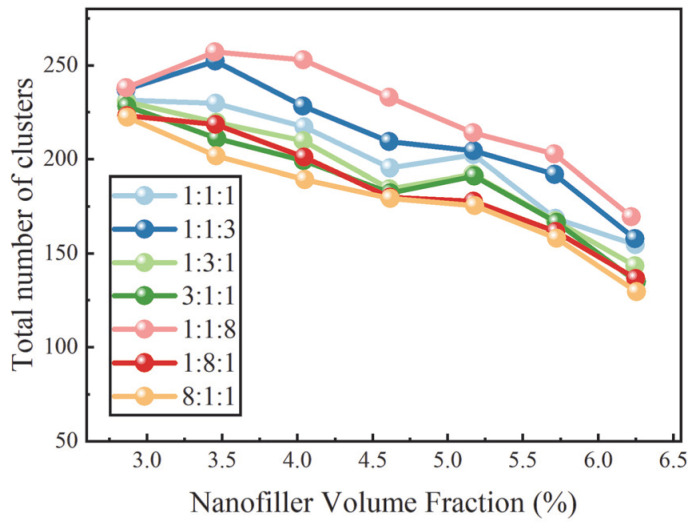
Dependence of the Nc on nanofiller VF for different filler composition ratios.

To further elucidate the influence of filler morphology ratios on the evolution of conductive network structures, Figure 5 and Figure 6 present the variations in the MCs and the Nc as functions of the filler VF, respectively. At a given filler VF, systems characterized by a larger dominant cluster and a smaller number of clusters exhibit stronger inter-filler connectivity and are therefore more likely to develop system-spanning conductive networks. As shown in Figure 5, MCs exhibits pronounced dependence on the filler composition ratio. Among all systems examined, the composition with a ratio of 8:1:1 consistently displays the largest dominant cluster across the investigated VF range. This behavior arises from the high aspect ratio of rod fillers, which enables them to act as effective bridging elements in space, rapidly merging local clusters into larger connected structures through extended geometric contacts and axially staggered arrangements. As the fraction of rod fillers decreases, their ability to construct and sustain large-scale networks is progressively weakened, leading to a marked reduction in MCs. This trend closely mirrors the corresponding evolution of the conductive probability, further confirming the critical role of rod fillers in promoting network connectivity.

By contrast, increasing the fraction of X fillers leads to pronounced network fragmentation, characterized by a significant decrease in the MCs and a concurrent increase in Nc. This behavior can be primarily attributed to the geometric characteristics of X fillers. Featuring a four-armed structure with nearly orthogonal arms, X fillers possess a more complex and strongly anisotropic excluded-volume shape, which makes it difficult for neighboring X fillers to identify stable and repeatedly occurring matching orientations in space. As a result, the formation of robust and extended contact chains is disfavored. Moreover, the geometry of X fillers also weakens their effectiveness as connectors that couple fillers of different morphologies into a unified network. Instead, X-rich systems tend to sustain multiple small- to intermediate-sized clusters rather than merging into a single dominant percolating cluster, thereby inhibiting the development of large-scale connectivity.

**Figure 7:**
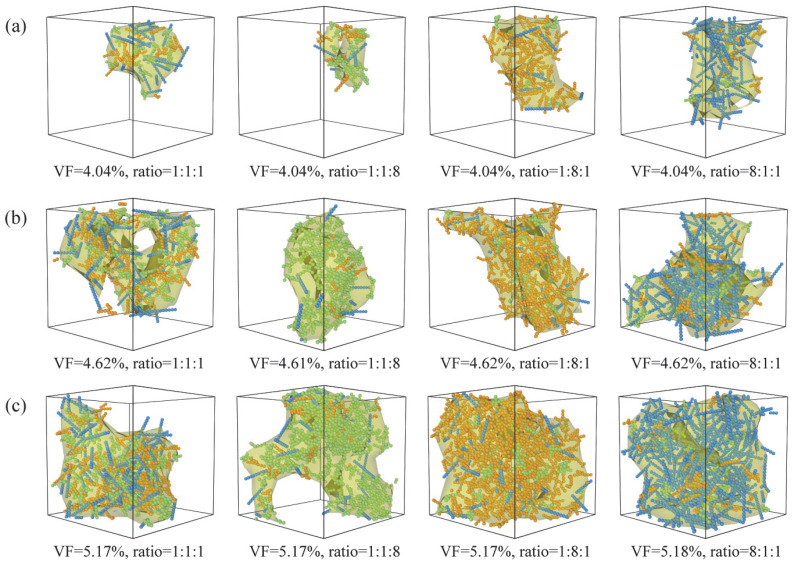
Representative configuration snapshots illustrating the evolution of conductive network structures for rod-rich, Y-rich, and X-rich systems at nanofiller VFs of 4.04%, 4.62%, and 5.17%. Rod, Y, and X nanofillers are shown in blue, yellow, and green, respectively. Representative configuration snapshots illustrating the evolution of conductive network structures for rod-rich, Y-rich, and X-rich systems at nanofiller VFs of (**a**) 4.04%, (**b**) 4.62%, and (**c**) 5.17%. Rod, Y, and X nanofillers are shown in blue, yellow, and green, respectively.

Figure 7 presents representative configuration snapshots of systems dominated by rod, Y, and X fillers, respectively, at nanofiller VFs of 4.04%, 4.62%, and 5.17%, illustrating the evolution of conductive network structures with increasing filler loading and varying morphology ratios. At the lower VF of 4.04%, the dominant clusters in all systems remain spatially limited, forming only several locally connected regions that are insufficient to span the simulation box. Consequently, the systems remain in a non-percolated state. As the VF increases to 4.62%, the conductive networks begin to develop significantly. Previously isolated local clusters gradually merge through the action of bridging fillers, leading to a marked increase in the size of the dominant cluster and the emergence of near-percolating network structures. At a VF of 5.17%, the conductive networks are essentially fully established. The dominant cluster spans the system continuously along all three orthogonal directions, forming a stable three-dimensional percolating network.

Distinct network morphologies observed at identical filler VF for systems dominated by different filler types are also clearly illustrated in Figure 7. When rod fillers constitute the majority, the system more readily develops a larger and more extended dominant cluster, resulting in a more continuous conductive backbone. In contrast, systems dominated by Y fillers exhibit dominant clusters of intermediate size, corresponding to a moderate level of network connectivity. For X-rich systems, the dominant clusters are noticeably smaller, reflecting a more fragmented network structure. These trends are fully consistent with the quantitative results obtained for the conductive probability, MCs, and Nc discussed, further highlighting the pronounced influence of filler morphology on network formation. Specifically, rod fillers, owing to their high aspect ratio, are more effective at constructing system-spanning conductive structures, whereas the geometric characteristics of X fillers favor the formation of dispersed and fragmented networks, making the attainment of three-dimensional percolation more difficult.

## 4. Conclusions

In this work, coarse-grained molecular dynamics simulations were employed to systematically investigate the effects of rod, Y and X nanofillers with varying composition ratios on the formation of conductive networks in polymer nanocomposites. By incorporating an efficient cKDTree-based neighbor search strategy, both the evolution of local filler structures and the resulting macroscopic percolation behavior were quantified with high computational efficiency and accuracy. The results demonstrate that filler morphology ratios play a critical role in determining the percolation threshold, and the overall topology of the conductive network.

At a fixed filler VF, nanofillers with different morphologies exhibit markedly distinct spatial organization and network-forming capabilities. Rod fillers, owing to their high aspect ratio, effectively promote connectivity across multiple length scales, leading to the formation of larger dominant clusters and a pronounced reduction in the percolation threshold. Y fillers, with their multidirectional contact geometry, contribute to enhanced local structural correlations but are insufficient to dominate long-range network percolation. By contrast, the complex geometry of X fillers reduces their connectivity efficiency, favoring the coexistence of multiple clusters and fragmented network structures, thereby increasing the percolation threshold. Statistical analyses of the MCs and the Nc further reveal the fundamental differences in network formation capabilities among the three filler morphologies. Higher rod filler fractions facilitate cluster merging and strengthen conductive networks, whereas X-rich systems exhibit enhanced cluster fragmentation and reduced network connectivity. The overall variation in the percolation threshold is therefore collectively determined by the synergistic and competitive interactions among fillers with different morphologies.

The morphology-controlled framework and the conductive probability analysis approach established in this study provide a new quantitative avenue for understanding charge transport mechanisms in multi-morphology nanofiller systems. The insights obtained herein offer theoretical guidance for optimizing filler combinations and morphology engineering in conductive polymer nanocomposites. Future studies may extend the present multi-morphology framework by incorporating external fields or working conditions to explore the cooperative effects between filler morphology and external stimuli on percolation behavior. In addition, integrating interfacial interactions and experimental validation will further broaden the applicability of this work and contribute to a more comprehensive theoretical foundation for the rational design of high-performance conductive composites.

## Figures and Tables

**Table 1:** VF corresponding to different numbers of nanofillers.

Number of Nanofillers	270	330	390	450	510	570	630
VF	2.87% ± 0.01%	3.46% ± 0.01%	4.04% ± 0.01%	4.62% ± 0.01%	5.17% ± 0.01%	5.72% ± 0.01%	6.23% ± 0.01%

## Data Availability

The original contributions presented in this study are included in the article. Further inquiries can be directed to the corresponding author.
